# Risk factors of deep vein thrombosis of lower extremity in patients undergone gynecological laparoscopic surgery: what should we care

**DOI:** 10.1186/s12905-021-01276-7

**Published:** 2021-03-26

**Authors:** Qing Tian, Meng Li

**Affiliations:** 1 Department of Operating Room, West China Second University Hospital, Sichuan University, Chengdu, China; 2 Key Laboratory of Birth Defects and Related Diseases of Women and Children, Sichuan University, Sichuan Province, No. 20, Section 3, Renmin South Road, Wuhou District, Chengdu, China; 3 Department of Gynaecology, West China Second University Hospital, Sichuan University, Chengdu, China

**Keywords:** Deep vein thrombosis, Gynecology, Laparoscopic surgery, Prevention, Treatment, Nursing care

## Abstract

**Background:**

Deep vein thrombosis (DVT) significantly influences the prognosis of patients. It’s necessary to analyze the risk factors for postoperative DVT in patients undergone gynecological laparoscopic surgery.

**Methods:**

Patients who underwent gynecological laparoscopic surgery from January 1, 2018 to May 31, 2020 were included. The characteristics and clinical data of DVT and non DVT patients were collected and analyzed. Logistic regression analysis was performed to identify the risk factors of DVT in patients undergone gynecological laparoscopic surgery.

**Results:**

A total of 355 patients undergone gynecological laparoscopic surgery were included, the incidence of postoperative DVT was 11.55%. There were significant differences in the age, hypertension, D-dimer, duration of surgery, intraoperative pneumoperitoneum pressure, duration of days in bed between DVT and non-DVT groups (all *p* < 0.05), and there were no significant difference in the BMI, diabetes, hyperlipidemia, ASA classification and intraoperative blood transfusion between DVT and non-DVT groups (all *p* > 0.05). Age > 50 years (OR 4.246, 95% CI 1.234–7.114), hypertension (OR 2.219, 95% CI 1.153–4.591), D-dimer > 0.5 mg/L (OR 3.914, 95% CI 1.083–5.229), duration of surgery ≥ 60 min (OR 2.542, 95% CI 1.101–4.723), intraoperative pneumoperitoneum pressure ≥ 15 mmHg (OR 3.845, 95% CI 1.119–5.218), duration of days in bed > 3 days (OR 1.566, 95% CI 1.182–1.994) was the independent risk factors for DVT in patients undergone gynecological laparoscopic surgery (all *p* < 0.05).

**Conclusions:**

The incidence of postoperative DVT in patients undergone gynecological laparoscopic surgery is high, and those high-risk factors should be targeted to intervene in order to reduce the postoperative DVT.

## Background

In recent years, with the continuous improvement of surgical technology and the improvement of surgical instruments and equipment, deep vein thrombosis (DVT) of the lower extremities is still one of the common complications after gynecological surgery [1]. The feared complication of DVT is pulmonary embolism, which is closely associated with higher mortality and poorer prognosis [2]. Furthermore, DVT produce adverse impact on the limb motor function of patients, thereby influencing the daily activity ability and reducing the quality of life [3]. Therefore, the preventions and treatments of DVT are on the top research agenda of current studies.

Compared with traditional laparotomy, gynecological laparoscopic surgery has less damage to the patient’s body, and the stress response is less, the patient loses less blood, and the postoperative recovery is faster [4]. However, DVT is a serious complication of laparoscopic surgery, which may be associated with intraoperative pneumoperitoneum pressure and other influencing factors [5]. It’s been reported that the risk of DVT after surgery is not lower than that of traditional gynecological surgery [6]. Therefore, it is very necessary to understand the related risk factors of DVT after gynecological laparoscopic surgery, to guide the early prevention and intervention of DVT in clinic, and to reduce the occurrence of DVT after surgery [7]. In this study, we aimed to analyze the relevant clinical data of patients undergone laparoscopic surgery, to explore the related factors of postoperative DVT, thereby providing evidence for clinical preventions and treatments of postoperative DVT in patients with gynecological laparoscopic surgery.

## Methods

### Ethical approval

Our study has been verified and approved by the ethics committee of West China Second University Hospital, Sichuan University (No.20180147), and written informed consents have been obtained from all the included patients. All the methods were performed in accordance with the relevant guidelines and regulations.

### Patients

Patients who underwent gynecological laparoscopic surgery from January 1, 2018 to May 31, 2020 were included as potential participants. The inclusion criteria for patients were: (1) the patients were underwent gynecological laparoscopic surgery for the first time; (2) all patients underwent venous color ultrasound to detect the DVT before and after surgery; (3) patients who did not use contraceptives or other hormonal drugs, or drugs that might affect the body's blood clotting function before surgery; (4) patients were well informed and agreed to participant in this study. The exclusion criteria for patients were: (1) Patients who received traditional gynecological open surgery; (2) Patients taking drugs for anticoagulation before surgery; (3) Patients suffering from diseases with abnormal blood coagulation function, such as hemophilia, leukemia et al.; (4) Patients who were not willing to participate in this study.

### Diagnosis of DVT

The diagnosis of DVT referred to the relevant diagnostic guidelines [8, 9]. The criteria for the diagnosis of DVT included: (1) Clinical manifestations: postoperative pain, numbness, swelling, and limited mobility of the affected side of the lower limb. (2) Signs: The circumference of the lower limb of the affected side was larger than that of the healthy side. The Homans sign was positive. (3) Auxiliary examination: During the color Doppler ultrasound venous examination of the lower extremities, there was substantial echo in the venous cavity.

### DVT prophylaxis

We routinely utilized the mechanical post-operative DVT prophylaxis for all included patients. We encouraged patients to get out of bed as soon as possible and to massage and observe their lower limbs frequently. All the patients underwent electrical stimulation for total of 3 days with Phcnix 200 massager (Guanger biotechnology, Shanghai, China) to start within 12 h after surgery. The first stage for the first two postoperative days: frequency 20/40/20, pulse width 370/330/370; Second stage for the third day: frequency: 65/105/65; Pulse width: 420/380/420, 30 min each time. The intensity of stimulation was different according to the tolerance of each patient, and it was set to the maximum intensity that could be tolerated by patients without discomfort.

### Data collection

The general patients’ information and related clinical data of each included patient were collected and analyzed, which including: patients’ age, body mass index (BMI), concurrent complications (diabetes, hypertension, hyperlipidemia), D-dimer value, American Association of Anesthesiologists (ASA) score, duration of surgery, intraoperative blood transfusion, intraoperative pneumoperitoneum pressure, duration of days in bed (the duration of daily bed stay ≥ 20 h). All the data were collected and recorded by two authors independently.

### Statistical analysis

This study used SPSS 21.00 statistical software package for statistical processing. The data was expressed in terms of rate. Firstly, single factor analysis was used, and the chi-square test was used to screen out the potential variables, and then multi-factor unconditional binary classification was used. Logistic regression analysis was performed to determine the odds ratio (OR) value of each risk factor and its 95% confidence interval (95% CI), and *p* < 0.05 was considered statistically significant.

## Results

### Included patients

A total of 355 patients undergone gynecological laparoscopic surgery were included. Of the 355 included patients, 41 patients were diagnosed as DVT after surgery, the incidence of DVT in patients undergone gynecological laparoscopic surgery was 11.55%. The distribution of types of gynecological laparoscopic surgery was presented in Table 1.

**Table 1:** Distribution of types of gynecological laparoscopic surgery

Surgery	Total cases	DVT	Incidence (%)
Gynecological accessory surgery	190	22	11.58
Myomectomy	79	9	11.39
Laparoscopic total hysterectomy	68	8	11.76
Laparoscopic gynecological malignant tumor surgery	18	2	11.11
Total	355	41	11.55

### The characteristics of included patients

As showed in Table 2, there were significant differences in the age, hypertension, D-dimer, duration of surgery, intraoperative pneumoperitoneum pressure, duration of days in bed between DVT and non-DVT groups (all *p* < 0.05), and there were no significant difference in the BMI, diabetes, hyperlipidemia, ASA classification and intraoperative blood transfusion between DVT and non-DVT groups (all *p* > 0.05).

**Table 2:** The characteristics of included patients

Variables	DVT group (n = 41)	Non-DVT group (n = 314)	χ^2^	*p*
Age (years)				
< 50	7 (17.07%)	227 (72.29%)	1.209	0.031
≥ 50	34 (82.93%)	87 (27.71%)		
BMI (kg/m^2^)				
< 25	26 (63.41%)	186 (59.24%)	1.144	0.101
≥ 25	15 (36.59%)	128 (40.76%)		
Diabetes				
Yes	12 (29.27%)	73 (23.25%)	1.118	0.062
No	29 (70.73%)	241 (76.75%)		
Hypertension				
Yes	22 (53.65%)	62 (19.75%)	1.133	0.019
No	19 (46.35%)	252 (80.25%)		
Hyperlipidemia				
Yes	10 (24.39%)	62 (19.76%)	1.181	0.112
No	31 (75.61%)	246 (78.34%)		
D-dimer (mg/L)				
< 0.5	5 (12.20%)	211 (67.20%)	1.226	0.036
≥ 0.5	36 (87.80%)	103 (32.80%)		
ASA classification				
Level I	20 (48.78%)	156 (49.68%)	1.140	0.074
Level II	21 (51.22%)	158 (50.32%)		
Duration of surgery (min)				
< 60	11 (26.83%)	181 (57.64%)	1.183	0.019
≥ 60	30 (73.17%)	133 (42.26%)		
Intraoperative blood transfusion				
Yes	10 (24.39%)	35 (11.15%)	1.128	0.054
No	31 (75.61%)	279 (88.85%)		
Intraoperative pneumoperitoneum pressure (mmHg)				
< 15	16 (39.02%)	202 (64.33%)	1.102	0.006
≥ 15	25 (60.98%)	112 (35.67%)		
Duration of days in bed				
< 3	12 (29.27%)	226 (71.97%)	1.177	0.031
≥ 3	29 (70.73%)	88 (28.03%)		

### The risk factors for DVT

We included age, hypertension, D-dimer, duration of surgery, intraoperative pneumoperitoneum pressure, duration of days in bed for further logistic regression, and the variable assignment of multivariate logistic regression was presented in Table 3. And as indicated in Table 4, Age > 50 years (OR 4.246, 95% CI 1.234–7.114), hypertension (OR 2.219, 95% CI 1.153–4.591), D-dimer > 0.5 mg/L (OR 3.914, 95% CI 1.083–5.229), duration of surgery ≥ 60 min (OR 2.542, 95% CI 1.101–4.723), intraoperative pneumoperitoneum pressure ≥ 15 mmHg (OR 3.845, 95% CI 1.119–5.218), duration of days in bed > 3 days (OR 1.566, 95% CI 1.182–1.994) was the independent risk factors DVT in patients undergone gynecological laparoscopic surgery (all *p* < 0.05).

**Table 3:** The variable assignment of multivariate logistic regression

Factors	Variables	Assignment
VTE	Y	Yes = 1, no = 2
Age	X_1_	≥ 50 = 1, < 50 = 2
Hypertension	X_2_	Yes = 1, No = 2
D-dimer	X_3_	≥ 0.5 = 1, < 0.5 = 2
Duration of surgery	X_4_	≥ 60 = 1, < 60 = 2
Intraoperative pneumoperitoneum pressure	X_5_	≥ 15 = 1, < 15 = 2
Duration of days in bed	X_6_	≥ 3 = 1, < 3 = 2

**Table 4:** The results of logistic regression analysis on the risk factors of DVT in patients with gynecological laparoscopic surgery

Variables	β	${\text{S}}\overline{{\text{x}}}$	OR	95% CI	*p*
Age > 50 years	0.833	0.213	4.246	1.234–7.114	0.012
Hypertension	0.914	0.240	2.219	1.153–4.591	0.028
D-dimer > 0.5 mg/L	0.871	0.251	3.914	1.083–5.229	0.008
Duration of surgery ≥ 60 min	0.624	0.118	2.542	1.101–4.723	0.031
Intraoperative pneumoperitoneum pressure ≥ 15 mmHg	0.649	0.104	3.845	1.119–5.218	0.022
Duration of days in bed > 3 days	0.213	0.124	1.566	1.182–1.994	0.037

## Discussions

At present, there are large differences in reports on the incidence of DVT after gynecological diseases. The incidence of DVT after gynecological surgery ranges from 11 to 29% [1, 10]. Sakon et al. [11] have pointed out that DVT occurred after gynecological surgery, the incidence of DVT can be as high as 17.5%. Domestic studies by Huang et al. [12] have shown that the incidence of DVT after gynecological laparoscopic surgery is 7.6%. In addition, several studies [13, 14] have shown that patients undergone gynecological laparoscopic surgery have significant increased postoperative blood viscosity, the incidence of DVT is about 8%. The incidence of DVT in this present study is 11.55%. The reasons for the differences in these reports may be that the design standards of various investigations are inconsistent, and the lack of multi-center or large-sample series of studies [15]. Furthermore, the operation techniques and methods of gynecological laparoscopic surgery in each hospital are not consistent [16]. Besides, a considerable number of postoperative DVT patients in clinic have no typical symptoms and signs, which brings certain difficulties to clinical DVT diagnosis [17]. Therefore, the incidence of DVT after gynecological laparoscopic surgery is high, and it is necessary to take preventive and intervention measures as soon as possible to reduce the incidence of DVT. The results our study have found that Age > 50 years, hypertension, D-dimer > 0.5 mg/L, duration of surgery ≥ 60 min, intraoperative pneumoperitoneum pressure ≥ 15 mmHg, duration of days in bed > 3 days the independent risk factors DVT in patients with gynecological laparoscopic surgery, it’s necessary to conduct many counteractive or preventative measures targeted on those factors to reduce the occurrence of DVT.

Many published studies [18, 19] have shown that the incidence of first DVT increases exponentially with age. Some studies [20, 21] have found that the annual incidence of DVT in children younger than 15 is less than 0.005%, but the annual incidence of DVT in adults over 80 years old increased significantly to 0.5%. The main reasons for the high incidence of DVT in elderly patients may be explained as follows: with the increase of age, the activities of elderly patients reduce accordingly, and the blood flow speed in the veins slows down, resulting in less and slower blood flow in the veins of the lower limbs [22, 23]. Besides, older women often have hypertension, hyperlipidemia, diabetes, etc. These complications can increase the blood viscosity [24]. In addition, with the increase of age, the elasticity of blood vessel walls in elderly patients decreases and the vascular endothelium is easily destroyed [25]. When endothelial cells release more procoagulant substances, the risk of DVT will increase accordingly [26]. Hypertension is a risk factor for DVT. Most patients with hypertension have dysfunction of the renin–angiotensin–aldosterone system, water and sodium retention are increased with more interstitial fluid and decreased plasma content in blood vessels, resulting in increased blood viscosity [27]. The function of vascular endothelial cells in patients with hypertension is disordered, resulting in the production of more oxygen free substance, inactivating more vasodilators, forming vascular inflammation, activating the blood coagulation system, and promoting the occurrence of DVT [28].

Increased D-dimer level is currently considered to be an effective indicator of abnormal blood coagulation system. There are reports [29, 30] showing that elevated D-dimer is of great significance in identifying lower extremity arterial thrombosis in peripheral diseases. Studies [31, 32] have reported D-dimer for the prediction of the occurrence of DVT in elderly hospitalized patients, the critical value ≥ 0.5 mg/L has good predictive value, with a sensitivity of 100% and a specificity of 41. 8%. The pneumoperitoneum pressure in gynecological laparoscopic surgery is generally about 12–15 mmHg, while the normal value of the human inferior vena cava pressure is 2–5 mmHg [33]. The greater the pneumoperitoneum pressure, the greater the pressure on the inferior vena cava, causing slow blood flow [34]. Once the blood becomes a hypercoagulable state, it can also cause endothelial damage and induce platelet activation adhesion and release, increasing the risk of VDT [35].

115 patients in our study had been bedridden for > 3 days. Although we encourage patients to get out of bed as soon as possible after surgery, most patients still choose to stay in bed. The reason may be related to the patient's fear of pain. We have not use analgesics on the patient after the operation. At the same time, many patients worry that getting out of bed too early will increase wound tension and delay wound healing. When the patient is bedridden for a long time, and the blood flow is slow, thereby the probability of VDT in elderly patients increases significantly. Patients who get out of bed early after surgery can increase muscle contraction, increase blood flow in the lower limbs, and reduce the occurrence of DVT [36, 37].

Pharmacological DVT prophylaxis not considered in our study because our pharmacological DVT prophylaxis is not a routine practice in our department. However, pharmacological DVT prophylaxis is a important issue that merits discreet consideration. The treatment of venous thrombosis mainly includes anticoagulant drugs such as low-dose heparin, low-molecular-weight dextran, and warfarin sodium. Low-dose heparin has little effect on the fetus during pregnancy, but it may cause thrombocytopenia, bleeding and osteoporosis in the body [38]. Low-molecular-weight dextran is usually used before and during surgery, but for patients over 40 years old, its effect in preventing postoperative venous thrombosis is not obvious [39]. As the vitamin K antagonist, warfarin sodium has been widely used in the anticoagulation treatment of various diseases. It can also be used in the treatment of patients with thrombosis or pulmonary embolism after gynecological surgery, but the use of warfarin sodium at any stage of pregnancy may lead to the abnormal development of the central nervous system of the fetus and neonatal bleeding, so warfarin sodium should be avoided as much as possible during pregnancy [40]. The blood coagulation function must be tested during the period of oral anticoagulant drugs [41]. Still the types and doses of pharmacological DVT prophylaxis need further investigations in the future studies.

It is necessary to take active measures to prevent the occurrence of DVT after gynecological surgery. A large number of studies [42, 43] have shown that the use of drug preventive measures can reduce the incidence of venous thromboembolism by 40% to 60%. At present, clinical recommendations for patients should be based on the risk factors for venous thromboembolism, it’s possible to give preventive treatment with low molecular weight heparin, unfractionated heparin or fondaparinux [44]. For patients with potential bleeding risk or severe bleeding, mechanical prevention, gradient compression stockings or intermittent inflation compression can be given [45]. Clinically, attention should be paid to the risk of DVT in elderly patients, shorten the operation time and postoperative bedridden time, and special attentions are highlighted to the activities of limb function. It is worth noting that the sample size of cases selected in this study is small and our study is a single-center study. The results of the study should be treated with caution. Future studies with larger samples and multi-centers need to further explore the risk factors of DVT in patients with gynecological laparoscopic surgery, to provide reliable evidence to the prophylaxis of DVT.

## Conclusions

In conclusion, for gynecological laparoscopic surgery patients with age > 50 years, hypertension, D-dimer > 0.5 mg/L, duration of surgery ≥ 60 min, intraoperative pneumoperitoneum pressure ≥ 15 mmHg, duration of days in bed > 3 days, the risk of DVT is higher, the management of such high-risk factors should be strengthened in clinical practice to reduce the incidence of DVT.

## Data Availability

All data generated or analyzed during this study are included in this published article.

## Abbreviations

DVT: Deep vein thrombosis
BMI: Body mass index
ASA: American Association of Anesthesiologists
OR: Odds ratio
95% CI: 95% confidence interval
